# Heterologous COVID-19 Booster Vaccination in the Chronic Disorder of Consciousness: A Pilot Study

**DOI:** 10.3390/clinpract12030037

**Published:** 2022-05-11

**Authors:** Maria Elena Pugliese, Riccardo Battaglia, Maria Girolama Raso, Raffaela Chiaravalloti, Francesco Coschignano, Angela Pagliuso, Roberta Bruschetta, Giovanni Pugliese, Paolo Scola, Paolo Tonin, Antonio Cerasa

**Affiliations:** 1 Intensive Rehabilitation Unit, S’Anna Institute, 88900 Crotone, Italy; me.pugliese@isakr.it (M.E.P.); r.battaglia@isakr.it (R.B.); m.raso@istitutosantanna.it (M.G.R.); r.chiaravalloti@isakr.it (R.C.); f.coschignano@isakr.it (F.C.); a.pagliuso@isakr.it (A.P.); g.pugliese@isakr.it (G.P.); p.scola@isakr.it (P.S.); patonin18@gmail.com (P.T.); 2 Institute for Biomedical Research and Innovation (IRIB), National Research Council of Italy, 98164 Messina, Italy; roberta.bruschetta@irib.cnr.it; 3 Pharmacotechnology Documentation and Transfer Unit, Preclinical and Translational Pharmacology Department of Pharmacy, Health Science and Nutrition, University of Calabria, 87036 Rende, Italy

**Keywords:** COVID-19, disorder of consciousness, vaccination, antibody responses, booster

## Abstract

Significant anti-spike protein receptor-binding domain (S-RBD) antibody responses have been demonstrated in patients with chronic disorder of consciousness (DOC) completing a COVID-19 vaccine regime with BNT162b2 (Pfizer–BioNTech). We now provide further prospective data on the immunogenicity of these patients followed by heterologous booster injection with mRNA-1273 (Moderna). These patients were compared with two different demographically comparable healthcare workers (HCW) groups who underwent homologous booster injection with BNT162b2 vaccine or heterologous booster injection with mRNA-1273. Antibody responses were evaluated at 21 days after the administration of the booster dose of vaccination. Results: No severe adverse reactions were reported after each type of vaccination. Heterologous boosting with mRNA-1273 elicited a higher increase of S-RBD IgG levels than homologous boosting with BNT162b2 both in DOC patients and HCW who had previously received two doses of BNT162b2. No significant difference was detected between DOC and HCW patients who received heterologous boosting. Conclusions: Despite the small sample size, our preliminary results suggest that heterologous boosting with mRNA-1273, following initial vaccination with BNT162b2, is safe and tends to be more immunogenic than homologous boosting, either in fragile people or in healthy controls.

## 1. Introduction

A COVID-19 booster dose has become mandatory after the demonstration that protection against SARS-CoV-2 infection waned after a two-dose schedule of vaccines, mainly to protect the most vulnerable patients. Rapidly increasing data have been provided comparing immunogenicity and safety of different third (booster) doses of COVID-19 vaccines [1,2,3,4,5,6]. Preliminary data have shown that receipt of a third dose that does not match (heterologous booster) the primary vaccination schedule could be considered in populations with a limited number of vaccines or in vaccinated populations where the immune response rate has waned over time. Atmar et al. [4] demonstrated that heterologous and homologous booster vaccines were immunogenic in adults who had completed a primary COVID-19 vaccine regimen and had an acceptable safety profile. Again, the UK COM-COV trial indicated that a heterologous prime-boost schedule can be more immunogenic than a homologous schedule [5]. However, there is a paucity of data concerning the frail populations at high risk of developing severe complications if infected by the COVID-19 virus. The efficacy and safety of heterologous booster vaccination in this category of patients was only evaluated in small groups of immunocompromised patients [7,8].

A vegetative state and minimally conscious state are disorders of consciousness (DOC) characterized by a severe neurological condition with acute and reversible or chronic and irreversible alterations in the level of consciousness. Despite it being well known that central nervous system injury leads to secondary immunodeficiency—CNS injury-induced immunodepression (CIDS)—and infection [9,10,11,12], to date, the long-term effects of brain injury on the immune system are largely unknown. Munno et al. [13] showed a profound impairment of phagocytosis and killing of monocytes in 14 vegetative state patients. In another study, Satzbon et al., investigated 11 post-traumatic persistent vegetative-state patients, finding an alteration of the humoral immunity, with a consequently defective opsonization and a neutrophil dysfunction in 27% of patients [14]. To date, the long-term effects of brain injury on the immune system are unknown and few data are available on DOC immunocompetence [13,14,15]. Our group has recently demonstrated the efficacy of seroconversion in DOC patients after vaccination with two doses of BNT162b2 mRNA vaccine, which significantly declines over time with respect to healthy individuals [16]. Now, we seek to evaluate how immunogenicity changes followed by heterologous boosting with mRNA-1273 at a dose of 50 mcg.

## 2. Materials and Methods

### 2.1. Enrollment

This study is a follow-up analysis conducted on a database used in a previous study [13], with additional data acquired overtime. In this second part, eight DOC patients were lost because of (a) discharge, (b) booster dose deferral due to clinical instability, or (c) death. Moreover, 10 healthcare workers (HCW) dropped out because of COVID-19 infection or vaccination with a different molecule of vaccine (in relation to the local availability). Thus, with respect to previous study [13], 24 DOC patients and 28 HCW were followed and enrolled in this new study after booster vaccination. To evaluate the different effects among booster vaccines, an additional group of 14 HCW with no previous history of neurological, psychiatric, or immunological diseases was further enrolled. These additional healthy controls were recruited by internal local advertisements from our clinic site in S’Anna Institute (Crotone, Italy).

Eligible participants were individuals who had completed the primary COVID-19 vaccine regimen with two doses of BNT162b2 mRNA vaccine, administered at the same time, and who had reported no history of SARS-CoV-2 infections. Exclusion criteria were as follows: (a) deferring the booster vaccination due to clinical instability (i.e., severe infections), (b) medically confirmed COVID-19 infection before and during protocol, and (c) adverse reaction to the vaccine or discharge/death. All individuals enrolled in this study were monitored with a monthly antigenic nasal swab for screening purposes.

### 2.2. Ethics

The study was approved by the Ethical Committee of the Calabria Region (N protocol n. 166, 15 July 2021), according to the Helsinki Declaration. Written informed consent for study participation, blood withdrawal, and data collecting was obtained from the legal representative of each patient and from HCW.

### 2.3. Procedure

This is a single-center and non-randomized study. All subjects (DOC and both groups of controls) completed the primary COVID-19 vaccine regimen with two doses of BNT162b2 vaccine, 21 days apart. The vaccination campaign started in January 2021. All subjects (both patients and HCW) were vaccinated at the same time, with the first dose (BNT162b2) on 16 January 2021, the second dose (BNT162b2) on 6 February 2021, and the third dose (BNT162b2 or mRNA-1273) between 11 and 18 November 2021.

#### End Points

In this new study we sought to evaluate (1) the waning of humoral immune response to primary COVID-19 vaccine cycle with BNT162b2 at 9 months in DOC vs. HCW, (2) the reactogenicity mRNA-1273 and BNT162b2 booster dose in HCW and in DOC, and (3) the 21-day humoral response of booster vaccination comparing heterologous and homologues vaccination strategies and responses in DOC vs HCW.

According to the National Medicine Agency in Italy (AIFA; https://www.aifa.gov.it/en/, accessed on 12 December 2021) and following recent evidence about the benefit of heterologous booster vaccination [2,3,4], DOC patients received the third dose of mRNA-1273 (DOC__heter_). Otherwise, due to the pandemic health crisis, supply chain delay, and local vaccine availability, the healthy controls were divided into two vaccination groups: 28 controls enrolled in our previous study [16] receiving a third dose of BNT162b2 vaccine (homologous vaccination) (HCW__homol_), and an additional14 controls receiving an heterologous (HCW__heter_) boosting dose of mRNA-1273 (as the DOC group).

Anti-S-RBD IgG levels were measured at the same time: 30 days (t0), 6months (t1), and 9 months (t2) after completing the vaccination cycle with BNT162b2 vaccine (DOC__heter_ and HCW__homol_), and then 21 days apart for the boosting dose (in all the three groups DOC__heter_, HCW__heter,_ HCW__homol_). Boosting intervention was made following EMA criteria (https://www.ema.europa.eu/en/news/comirnaty-spikevax-ema-recommendations-extra-doses-boosters. accessed on 12 December 2021).

The laboratory staff as well as clinicians assessing adverse effects were blinded to the treatment assignments. As the vials and syringes for mRNA-1273 and BNT162b2 were different, additional unblinded personnel were responsible for vaccine preparation and administration. This unblinded personnel did not participate in any other trial process. After the booster vaccination, all healthy controls were observed at the clinic for 30 min after vaccination and then were instructed to keep a daily record of adverse vaccine-associated reactions.

### 2.4. Biochemical Analysis

For biochemical analysis, 4 weeks, 6 months, and 9 months after the second vaccine dose, and again 21 days after the boosting dose, we dosed the antibody IgG anti-SarsCov19 titer (S1-RBD) in DOC__heter_ and HCW__homol_ groups. We also dosed the antibody IgG anti-SarsCov19 titer (S1-RBD) in healthy controls (HCW__heter_) receiving a boosting dose of mRNA-1273 as the DOC group.

In this study, a commercially available immunoassay was used, the anti-SARS-CoV-2 S-RBD IgG (Snibe Diagnostics, New Industries Biomedical Engineering Co., Shenzhen, China). SARS-CoV-2 S-RBD IgG is a chemiluminescent immunoassay (CLIA) that determines IgG Ab against the RBD of the Spike (S) protein of the virus, in human serum or plasma. All analyses were performed on Maglumi 800 (Snibe Diagnostics, Italy), with results expressed in BAU/mL (WHO 20/136). Negative results are depicted as 0.99 BAU/mL.

### 2.5. Statistical Analysis

Statistical analyses were performed using R Language v.4.0.3 (R Foundation for Statistical Computing, Vienna, Austria).

Differences in antibody responses were evaluated between patients (HCW__heter_) and controls (HCW__homol_) over the three timepoints preceding the booster vaccination (t0, t1, and t2). Moreover, differences after booster vaccination were also assessed among the three groups (DOC__heter_, HCW__homol,_ and HCW__heter_). We applied the log2 transformation to IgG antibodies response and checking normal distribution through the Shapiro–Wilk test. Independent *t*-test and one-way ANOVA were employed to evaluate differences between groups. All statistical analyses had levels of <0.05 for defining significance.

## 3. Results

The demographic of all participants and clinical characteristics of DOC patients are summarized in Table 1. The three groups were comparable for all variables, except for the prevalence of hypertension, which was more frequently recorded in DOC patients.

**Figure 1:**
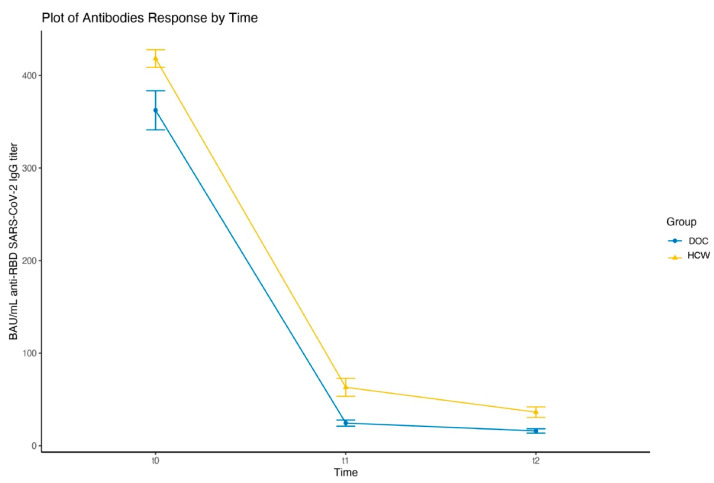
Mean antibody responses over the three timepoints in DOC patients and healthcare workers (HCW) after completion of regime vaccination with two doses of BNT162b2.

With respect to our previous study [13], we extended the analysis made at t0 (30 days) and t1 (180 days) after completion of regime vaccination with a further follow-up evaluation at 9 months. Overall, we confirmed that DOC patients are characterized by a significant decrease of antibody responses with respect to controls, which persists after 9 months (t = −3.14 *p*-level = 0.003). Figure 1 shows the distribution of antibody responses over the three timepoints in the two groups.

After the booster dose of vaccination, none of the DOC__heter_ and HCW individuals developed severe adverse effects. A high percentage of healthy subjects reported mild symptoms of local pain (79% HCW__homol_, 72% HCW__heter_) and fatigue (25% HCW__homol_, 29% HCW__heter_), whereas some developed fever (7% HCW__homol_, 15% HCW__heter_). In DOC patients, we could only observe and report objective adverse reactions (i.e., fever) or severe adverse reactions (respiratory distress, seizures, or death). Patients showed very few side effects, with only one patient with a high fever. We had no cases of respiratory distress, seizures, or death.

With respect to the t2 pre-booster phase, the third dose of vaccine increased S-RBD IgG levels by a factor of 2 to 114 for DOC__heter_ patients, and by a factor of 2 to 32 for HCW__homol_.

**Figure 2:**
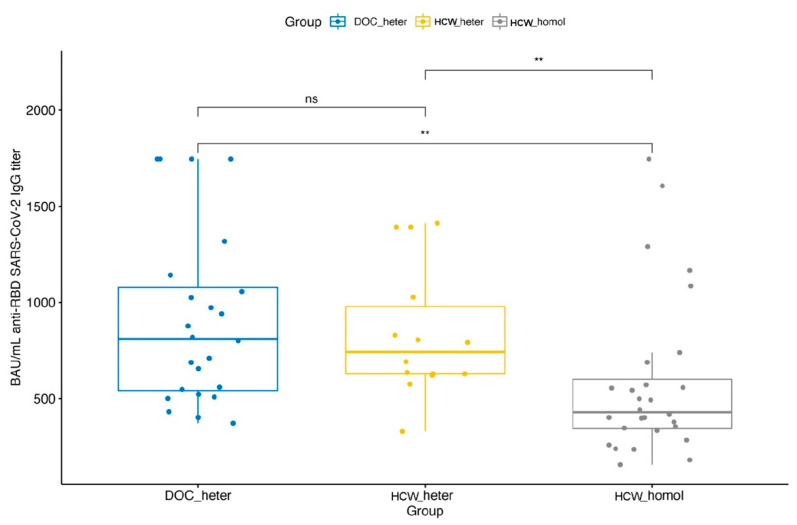
Pairwise comparisons of antibodies’ responses after the booster dose of vaccination between the study groups. ** significant at *p*-level < 0.05.

Heterologous boosting with mRNA-1273 elicited a higher increase of S-RBD IgG levels than homologous boosting with BNT162b2 in DOC patients and HCW who had previously received two doses of BNT162b2 (F = 7.42; *p*-level = 0.001). Post hoc analysis confirmed that healthy controls with homologous vaccination are characterized by the lowest antibodies response, either with respect to the other control group with heterologous vaccination (t = −2.77; *p*-level 0.022) or DOC patients (t = 3.54; *p*-level 0.002) (Figure 2). All previous analyses were rerun using age, gender, and the prevalence of hypertension as confounding variables. The overall pattern of findings remained the same. Table 2 summarizes the detected antibodies’ responses during all phases of this study.

## 4. Discussion

In our previous study [16], we demonstrated an efficient anti-SARS-CoV-2 S-RBD IgG antibody response in DOC patients completing a COVID-19 vaccine regime with BNT162b2, with a significantly greater decrease with respect to controls at 6 months. We concluded that this preliminary data could be helpful in assisting policymakers and practitioners when choosing the most effective additional vaccine doses for this at-risk category. Since levels of binding and neutralizing antibodies are related to vaccine efficacy for both adenovirus-vectored and messenger RNA (mRNA) vaccines, the measurement of these levels can help predict efficacy after boosting [17]. In this second follow-up study, we confirmed that DOC patients are characterized by a significant waning of the immune response over time (9 months), highlighting the importance of planning additional vaccine boosting doses. Indeed, the heterologous booster injection with mRNA-1273 elicited a higher increase of S-RBD IgG levels than homologous boosting with BNT162b2 in DOC patients as well as HCW, who had previously received two doses of BNT162b2.

Despite the obvious limitations related to the small size and the lack of an additional patient group undergoing homologous boosting vaccination, our data perfectly agree with previous studies [4,5,6,8] investigating healthy subjects. In particular, Munro et al., [6], with the COV-BOOST study group, analyzed the immunogenicity and safety in 2878 individuals of seven COVID-19 vaccines as a third dose (booster) following two doses of ChAdOx1 nCov-19 or BNT162b2. The best booster overall appears to be mRNA-1273 and the heterologous combination BNT/BNT/mRNA-1273. However, it is important to note that mRNA-1273 was also given at the highest dose (100 mcg) compared to other mRNA vaccines. In our study, instead, the used dose of mRNA-1273 was the half dose (50 mcg), as indicated by Public Health Control Service. Similar findings were found by Liu et al. [5] in 830 healthy controls after heterologous boosting with adenoviral-vectored (ChAdOx1 nCoV-19, AstraZeneca) or mRNA BNT162b2 vaccines.

### Limitations

The major limitations of the study are the small sample size, the impossibility to evaluate a further DOC group receiving homologous vaccination, and the lack of standardized procedure for assessing adverse effects in DOC patients.

## 5. Conclusions

Our pilot study suggests that the heterologous prime-boost regimen with the mRNA-1273 vaccine is safe and highly immunogenic for DOC patients. The high enhancement of antibody titers after heterologous boosting is notable, but the duration of these antibodies still requires further investigation, along with the neutralizing activity of these antibodies against other variants. Despite the data obtained in a small sample of HCW, this additional study might have the merit to move clinical interest to this frail population, although it should be important to further evaluate the impact of different incoming heterologous interventions.

## Figures and Tables

**Table 1:** Demographic and clinical characteristics of enrolled DOC patients and controls undergoing booster vaccination.

	DOC__heter_ (*n* = 24)	HCW__heter_ (*n* = 14)	HCW__homol_ (*n* = 28)	*p*-Level
Age	54.1 ± 16.5	44.3 ± 11.1	52.9 ± 12.5	n.s.
Gender, (%) male	55%	50%	53%	n.s.
Hypertension (Yes; %)	46%	7%	7%	<0.05
Diabetes mellitus (Yes; %)	0%	0%	3.50%	n.s.
Heart disease (Yes; %)	16%	3.50%	3%	n.s.
Renal insufficiency (Yes; %)	0%	0%	7%	n.s.
Obstructive pulmonary disease (Yes; %)	8%	0%	7%	n.s.
Liver disease (Yes; %)	8%	0%	0%	n.s.
Endocrinopathies (Yes; %)	12.50%	7%	7%	n.s.
Tumor (Yes; %)	2%	0%	0%	n.s.
**DOC-related Clinical Data**				
CRS-r at enrollment	9.2 ± 4.7			
Time from injury (years)	3.9 ± 3.3			
Etiology *n* (%)				
Vascular	11 (45.9%)			
Traumatic	6 (25%)			
Anoxic	4 (16.6%)			
Others	3 (12.5%)			
(Dementia, infections/post-surgery)				
Diagnosis *n* (%)				
VS	10 (41.6%)			
MCS	14 (59.4%)			

VS: vegetative state; MCS: minimally conscious state. CRS: Coma Recovery Scale—Revised. n.s.: not significant.

**Table 2:** Raw and transformed Log2 values of antibody responses in DOC and HCW groups.

	Pre-Booster Phase						Post-Booster Phase	
	t0 (1 Month)		t1 (6 Months)		t2 (9 Months)		21 Days	
	Raw	Log2 t0	Raw	Log2 t1	Raw	Log2 t2	Raw	Log2
DOC_heter	370.7 ± 106.4	8.4 ± 0.5	24.5 ± 17.8	4.2 ± 1.02	16 ± 12	3.7 ± 0.9	909.9 ± 453.9	9.6 ± 0.7
HCW_homol	419 ± 53.1	8.7 ± 0.2	65 ± 55.7	5.6 ± 1.1	36 ± 32.1	4.7 ± 1.1	584.9 ± 416.4	8.9 ± 0.9
HCW_heter							840.4 ± 340.3	9.6 ± 0.6

Data are shown in BAU/mL.

## Data Availability

The data presented in this study are available on request from the corresponding author.
